# Development and validation of the peptic ulcer scale under the system of quality of life instruments for chronic diseases based on classical test theory and generalizability theory

**DOI:** 10.1186/s12876-020-01562-y

**Published:** 2020-12-14

**Authors:** Chonghua Wan, Ying Chen, Li Gao, Qingqing Zhang, Peng Quan, Xiaoyuan Sun

**Affiliations:** 1 School of Humanities and Management, Research Center for Quality of Life and Applied Psychology, Key Laboratory for Quality of Life and Psychological Assessment and Intervention, Guangdong Medical University, 1 Xincheng Road, Songshan Lake Science and Technology Industry Park, Dongguan, 523808 Guangdong People’s Republic of China; 2 School of Public Health, Kunming Medical University, Kunming, 650500 People’s Republic of China; 3 Taian City Central Hospital, Taian, 271000 People’s Republic of China; 4 Department of Statistics and Medical Record Management, Shandong Provincial Hospital Affiliated To Shandong University, Jinan, 250021 People’s Republic of China

**Keywords:** Quality of life, Peptic ulcer, Standardized response mean, Psychometric properties, Generalizability theory

## Abstract

**Background:**

Quality of life (QOL) for patients with Peptic ulcer disease (PUD) is of interest worldwide and disease-specific instruments are needed for clinical research and practice. This paper focus on the development and validation of the PUD scale under the system of quality of life instruments for chronic diseases (QLICD-PU) by the modular approach and both classical test theory and Generalizability Theory.

**Methods:**

The QLICD-PU is developed based on programmatic decision-making procedures, including multiple nominal and focus group discussions, in-depth interviews, and quantitative statistical procedures. Based on the data of 153 PUD inpatients, correlation analysis, factor analysis, t-test, and Generalizability Theory analysis (including generalizability study and decision study, ie. G-study and D-study) were used to assess the validity, reliability, and responsiveness of the scale.

**Results:**

When the popular scale health survey short form (SF-36) was used as the standard, correlation and factor analysis confirmed good construct validity and criterion-related validity of QLICD-PU. Except for the social domain (0.62), the internal consistency α of all domains is higher than 0.70. The overall score and the test–retest reliability coefficients (Pearson r and intra-class correlation ICC) in all domains are higher than 0.80 (0.77 in the social domain). After treatments, the overall score and scores of all domains have statistically significant changes (*P* < 0.01), except for social impact and sexual function scores. The SRM (Standardized response mean) of domain-level scores ranges from 0.34 to 1.03. The G coefficient and reliability index (Ф coefficient) further confirm the reliability of the scale through more accurate variance components and decision-making information about changes in the number of items.

**Conclusions:**

The QLICD-PU can be used as a useful measurement to assess the quality of life of PUD patients with good psychometric characteristics and multiple advantages.

## Backgrounds

Peptic ulcer disease (PUD) is a frequently occurring and common disease in the world and is usually recurrent [1–5], with its annual incidence rate being 1.1–3.3% and the prevalence being 1.7–4.7%. About 10% of the people are suffered from this disease during their lifetime in the United States [1, 2], and also same proportion in Europe [4, 5]. Patients with PUD may have various gastrointestinal symptoms including abdominal pain, vomiting, and upper gastrointestinal bleeding which is related to high mortality and high morbidity [6, 7]. Considering the disease can result in many gastrointestinal symptoms such as pain, nausea, anorexia and some limitations to social and metal health, it is particularly important to evaluate their overall impact from the patient’s health-related quality of life (HRQOL) [8, 9]. Several studies showed that patients with PUD had significantly lower HRQOL than the general population and the improvement in HRQOL plays an important role in the treatment of the disease [9, 10]. It is hoped that the use of appropriate tools can improve the understanding of the treatment and service needs of PUD patients.

There are many HRQOL instruments which can be divided into general measures and specific measures against diseases. Contrast to the general measures focusing on comparisons of the results of different populations and interventions, the specific measures are more sensitive to detect and quantify subtle changes that are important to clinicians or patients [11]. For the last few decades, although the measurement of general QOL has been improved using the popular scale health survey short form (SF-36), PGWB (Psychological General Well-Being) index, etc., clinicians and researchers still need to determine the clinical significance of any measure of patients’ response to treatment. Therefore, several HRQOL measures against PU have been developed such as QPD (quality of life in peptic disease)[12], QLDUP (Quality of Life in Duodenal Ulcer Patients) [13]. Besides, the QOLRAD (Quality Of Life in Reflux and Dyspepsia) [14, 15], the FDDQL (Functional Digestive Disorder Quality of Life Questionnaire) [16] and the GSRS (Gastrointestinal Symptom Rating Scale) [17] can also be used for patients with PUD. Among them, the QLDUP (a 54 items questionnaire with 15 dimensions) was developed by combining the SF-36, PGWB index, and 13 disease specific items derived from patient and clinician interviews. The QOLRAD is a 25 items questionnaire with five subscores (each item scored on a seven point Likert scale).

However, these instruments are not developed based on the modular approach-a general/core module plus the specific modules [18, 19]. Considering that diseases within the same disease class (for example digestive diseases) have many characteristics (symptoms and side effects) in common, a popular approach to develop QOL instruments for diseases is to combine a general module for the entire class of diseases with the specific module for each individual disease. This method can greatly reduce the time and effort of developing new QOL scales. For instance, the quality of life questionnaires (QLQs) from EORTC (European Organization for Research and Treatment of Cancer) and the Functional Assessment of Cancer Therapy (FACTs) have been developed based on this modular principle [18, 19].

To the best of our knowledge, no instrument for PUD has been developed based on the modular approach, let alone a combination of classic test theory (CTT) and generalizability theory (GT). Therefore, we developed a set of Quality of Life Instruments for Chronic Diseases (QLICD) through a modular approach [20–23]. The system includes a general module (QLICD-GM) which can be used for all types of chronic diseases, as well as specific modules only for related diseases [20–23]. For example, the instrument QLICD-CHD for coronary heart disease is constructed by combining QLICD-GM with the specific module for coronary heart disease [21]. At present, the QLICD (V1.0) includes a 30-items general module QLICD-GM (3 domains and 10 facets) and 9 specific modules which form 9 specific scales of the QLICD-CHD [21], the QLICD-HY (hypertension) [22], the QLICD-IBS(irritable bowel syndrome) [23] and the QLICD-PU(peptic ulcer disease) etc.

In the current research, we aimed to develop and validate the QLICD-PU instrument.

## Methods

### Development of the QLICD-PU

The QLICD-PU consists of a general module QLICD-GM and a module dedicated to PUD. The development process of QLICD-GM has been described in another paper [20]. Here, we briefly summarize the development steps and results. The programmed procedures which include focus group discussions, in-depth interviews, pre-testing and four quantitative statistical analyses were used to select items. Finally, the QLICD-GM has 30 items which included 3 domains and 10 facets. The QLICD-GM has showed good psychometrics (reliability, validity, responsiveness) by the analysis from the data of 620 patients with seven kinds of chronic diseases such as coronary heart disease and hypertension [20].

For a specific module, 29 items reflecting symptoms and side-effects of PUD were selected to constitute the initial item pool. We selected these items from literature reviews and nominal / focus group discussions. Focus groups evaluate the importance of each item by ranking each item independently and then discussing the 9 lowest ranked items that are excluded. Consequently, the remaining 20 items constitute a preliminary questionnaire for conducting the pilot test and also Interviews with 29 PUD patients and 14 clinicians and researchers with extensive experience. We focus on patient opinion, which is most important for assessing the acceptability of interventions and related compliance. Based on the pilot data, the items were re-screened using similar development process to the generic module (statistical procedures and focus group discussion). The final specific module consists of 14 items coded PU1-PU14 (see Table 1 in detail), which can be classified into 6 facets.

**Table 1:** Correlation coefficients r among items and domains of QLICD-PU (n = 153)

Code	Items brief description in English	Physical	Psychological	Social	Specific
PH1	Take care of daily life (e.g. eating)?	**0.70**	0.14	0.34	0.06
PH2	Felt easily fatigued?	**0.65**	0.33	0.21	0.19
PH3	Have trouble walking 800 m or more?	**0.76**	0.12	0.24	0.04
PH4	Have trouble going up and down stairs?	**0.71**	0.32	0.20	0.04
PH5	Need to take medication?	**0.65**	0.33	0.23	0.19
PH6	A good appetite?	**0.49**	0.10	0.17	0.09
PH7	Satisfied with your sleep?	**0.55**	0.20	0.14	0.15
PH8	Felt pain or uncomfortable?	**0.52**	0.26	0.24	0.43
PS1	Memory and concentration affected?	0.39	**0.46**	0.40	0.16
PS2	Felt mentally miserable?	0.35	**0.59**	0.32	0.28
PS3	Felt lonely and helpless?	0.14	**0.69**	0.42	0.19
PS4	Felt pessimism and despair?	0.18	**0.72**	0.37	0.26
PS5	Worried about disease?	0.31	**0.72**	0.27	0.39
PS6	Felt fretful or irritable?	0.16	**0.56**	0.24	0.31
PS7	Felt nervous and anxious?	0.26	**0.68**	0.20	0.31
PS8	Stop medication because of side effects?	0.10	**0.44**	0.05	0.14
PS9	To be a burden to the family?	0.36	**0.55**	0.37	0.23
PS10	Felt self-abasement because of disease?	0.03	**0.69**	0.20	0.23
PS11	Hidden emotions but could not forget?	0.11	**0.68**	0.29	0.27
SO1	Interfered with work/housework?	0.29	0.35	**0.53**	0.18
SO2	Family roles?	0.16	0.03	**0.38**	0.10
SO3	Decreased caring and attention to family?	0.28	0.36	**0.34**	0.20
SO4	Good relations with family?	0.01	0.09	**0.56**	0.04
SO5	Help and support from family?	0.06	0.03	**0.52**	0.08
SO6	Affected participating in leisure activities?	0.18	0.35	**0.41**	0.07
SO7	Treat illness positively and optimistically?	0.23	0.30	**0.63**	0.23
SO8	Treatments received good for curing?	0.12	0.22	**0.35**	0.16
SO9	Economic problems caused by illness?	0.11	0.35	**0.48**	0.27
SO10	Support from friends and relatives?	0.13	0.01	**0.51**	0.06
SO11	Affected sexual activities?	0.27	0.27	**0.35**	0.16
PU1	Have pain (sore) in epigastria?	0.28	0.11	0.11	**0.53**
PU2	Have heartburn in epigastria?	0.08	0.17	0.14	**0.49**
PU3	Have pain/discomfort at night or hungry?	0.17	0.08	0.05	**0.51**
PU4	Pain/uncomfortable relieved after dinner?	0.13	0.00	0.07	**0.33**
PU5	Have acid regurgitation?	0.06	0.17	0.05	**0.48**
PU6	Have any belch (burps)?	0.19	0.12	0.08	**0.49**
PU7	Abdominal distension?	0.03	0.07	0.15	**0.53**
PU8	Salivate (flow saliva)?	0.06	0.26	0.00	**0.38**
PU9	Move bowels normal?	0.27	0.10	0.19	**0.34**
PU10	Upset/distress for gastroscopy inspection?	0.13	0.21	0.05	**0.36**
PU11	Vexed for food limit?	0.05	0.30	0.12	**0.43**
PU12	Troubled/limit by dine at fix time?	0.14	0.18	0.14	**0.35**
PU13	Worried about causing severe disease?	0.04	0.36	0.22	**0.51**
PU14	Vexed for often taking stomach medications?	0.01	0.31	0.17	**0.55**

Correlations between each item and its designated domain are in bold type

### Validation of the QLICD-PU

#### Data collection and scoring

In this study, we enrolled participants with PUD at any stage who were: (1) be able to provide written informed consent; (2) be able to read and write words with assistance. There were no protocol requirements regarding specific clinical treatment of patients. Physicians could treat the patients according to what they deemed clinically appropriate.

The survey was carried out at the First Affiliated Hospital of Kunming Medical University after approved by the ethics committee of this University. The respondents were voluntary and provided written consent for participation. Each interviewee was required to answer the questionnaire upon admission. Researchers including doctors and medical graduate students explained the purpose of the study and obtained informed consent before the test. The respondents were voluntary and provided written consent for participation.

To assess the reliability of the test–retest, a subsample is randomly selected for the second assessments on the second day of hospitalization. All patients available at the scheduled third evaluation time point have completed discharge measures to assess the responsiveness of the questionnaire.

Besides, the Chinese version of SF-36 [24] was also used to provide data for assessing the criterion-related validity, as well as convergent and discriminant validity of the QLICD-PU because of the lack of an agreed-upon gold standard for PUD. Baseline socio-demographic characteristics were recorded from hospital medical records, including age, gender, education level, marital status, clinical history, and treatment. Each investigator checked the answers immediately to ensure their integrity.

Since each item uses the five-point Likert format (not at all, a little bit, somewhat, quite a bit, and very much), positively stated items will be scored directly from 1 to 5, while negatively stated items will receive the opposite score. The domain/facet and overall scores are obtained by adding related item scores, all of which are linearly converted to standardized scores on a scale of 0–100. The higher the score of QLICD-PU means the better quality of life for both raw and standardized scores.

### Psychometric analysis

The validity, reliability, and responsiveness of QLICD-PU were evaluated in this study. The construct validity was evaluated by the Pearson correlation coefficient (r) between the items and the domains and also by factor analysis, while the criterion-related validity was assessed by correlating the corresponding domains of QLICD-PU and SF-36. Multi-trait scaling analysis [25] was used to test the convergence validity and discriminant validity. There are two validity criteria: (1) When the item-domain correlation is 0.40 or higher, it supports convergence validity; (2) discriminant validity is revealed when item-domain correlation is higher than that with other domains.

In terms of reliability, for each domain/facet and the overall scale, the internal consistency was assessed by Cronbach's alpha coefficients using the first measurement data (at admission) for large sample. Evaluation of test–retest reliability was by Pearson correlation coefficient and intra-class correlation (ICC) [26, 27] between the first and second assessments. The responsiveness (sensitivity to detect change) was assessed by using a paired t-test to compare the average score change between the two assessments before and after treatments and also the effect size, standardized response mean (SRM) [28, 29].

### Generalizability theory analysis

In addition to the classical test theory analysis, we also applied the Generalizability Theory (GT) in this research to study the reliability of the QLICD-PU score. GT is a modern test theory developed based on the combination of experiment design and analysis of variance. It is proposed as a method to improve measurement program design in an attempt to obtain reliable data [30–33]. To control the measurement errors, GT introduces independent variables or factors that interfere with test scores into measurement models, such as research objects, item difficulty, scoring criteria, and the interaction between these factors. An analysis of variance was then used to assess the impact of these variables or factors on test scores, using the variance component as an index. GT includes generalizability study (G-study) and decision study (D-study). G study quantified the amount of variance related to the different facets (factors) to be examined, while D study provides information about which protocol is best for a particular measurement by generating a generalizability (G) coefficient.

In our research, both G study and D study were completed in one measurement model to estimate the variance components and dependability coefficients in one-facet crossed design (person-by-item design, ie. p × i design). We defined the patient's quality of life as the measurement target and the item as a facet of measurement error. Specifically, we defined an acceptable observation range composed of measurement objects and measurement errors and estimated variance components for G-Study. And for D-study, we defined the allowable summary based on the measurement object and the measurement facet that the researchers are willing to summarize to express the measurement conditions. At the same time, the generalized coefficients of each facet and the variance components of the reliability indicators and their interactions were calculated.

## Results

### Socio-demographic characteristics of the sample

153 PUD patients range in age from 16 to 79, with a mean age of 45.2 ± 14.8. 110 cases (71.9%) were male, and 134 (87.6%) were of Han ethnicity. 27 cases (17.6%) finished primary school, while 85 (55.5%) completed high school, and 40 (26.2%) had a university or graduate degree. In terms of occupation, workers accounted for 38.6% (59 cases), farmer 15.0% (23),cadre 12.4% (19), teacher 9.2% (14), and others 24.8% (38). For perceived Income, poor accounted for 30.7% (49 cases), fair 58.8% (90), and high 9.2% (14).

### Construct validity

The construct validity was evaluated by item-domain Pearson’s correlation coefficient r and by factor analysis. A correlation analysis from the data measured at the time of admission showed that there is a strong correlation between the items and their domain (most above 0.40). However, the relationship between the item and other domains is weak ( see Table 1 in details). For example, the correlation coefficients between PHD and PH1-PH8 are between 0.49 and 0.76 (the first column in bold), which are higher than those between PHD and other items. Similarly, correlation coefficients between PSD and items of PS1- PS11 ranging from 0.44 to 0.72 (the second column in bold) are higher than the value between PSD and other items. Factor analysis was performed on the general module and the specific module respectively. After extraction standard was set as criteria of eigenvalues > 1, there were 8 principal components extracted from 30 items of the general module (QLICD-GM), accounting for 63.88% of the cumulative variance. By using the Varimax rotation method, it can be seen that the 8 principal components reflected 8 different facets under three domains of the general module with the first, fourth and fifth principals components mainly representing the psychological domain with higher loadings on PS1-PS11; the second and seventh principal components largely reflecting the physical domain with higher loadings on PH1-PH8; the third, sixth and eighth principal components generally depicting the social domain with higher loadings on SO1-SO11. Similarly, the principal component factor analysis extracted 6 principal components from the 14 items of the specific module with the cumulative variance of 65.88%, reflecting 6 facets.

### Criterion-related validity

The correlation coefficients between the QLICD-PU and SF-36 domain scores were listed in Table 2, indicating that the correlation between the same and similar domains (bold in the table) is usually higher than different and dissimilar domains. For example, the coefficient between the physical domain of QLICD-PU and the physical function of SF-36 is 0.67, which is higher than any other coefficient in this row. Similarly, the coefficient between the psychological domain of QLICD-PU and the mental health of SF-36 is 0.51, higher than any other coefficient in this row.

**Table 2:** Correlation Coefficients among domain scores of QLICD-PU and SF-36 (n = 153)

QLICD-PU	SF-36									
	PF	RP	BP	GH	VT	SF	RE	MH	PCS	MCS
PHD	**0.67**	0.26	0.26	0.37	0.31	0.18	0.16	0.19	0.61	0.30
PSD	0.23	0.21	0.21	0.40	0.30	0.38	0.27	**0.51**	0.38	0.51
SOD	0.37	0.20	0.31	0.47	0.33	**0.54**	0.23	0.31	0.53	0.47
SPD	0.14	0.18	**0.33**	0.29	0.12	0.24	0.20	0.23	0.31	0.25
TOT	0.49	0.30	0.39	0.56	0.37	0.39	0.31	0.49	**0.64**	**0.54**

Correlations between same/similar domains were presented in bold type

PHD: physical domain, PSD: psychological domain, SOD: social domain, SPD: specific domain, TOT: total score

PF: physical function, RP: role-physical, BP: bodily pain, GH: general health, VT: vitality, SF: social function, RE: role-emotional, MH: mental-health, PCS: Physical Component Summary, MCS: Mental Component Summary

### Reliability

As shown in Table 3, the Cronbach's α for these four domains were higher than 0.70 except for SOD (0.62), while they were ranging from 0.35 to 0.81 at facets level.

**Table 3:** Reliability of the quality of life instrument QLICD-PU (n = 153 for *α*, n = 63 for *r* and ICC)

Domains/facets (number of items)	Internal consistency coefficient *α*	Test–retest coefficient *r*	Test–retest ICC (95% CI)
Physical function PHD (8)	0.79	0.89	0.89 (0.83–0.93)
Independence (3)	0.81	0.88	0.88 (0.82–0.93)
Appetite and Sleep (2)	0.49	0.74	0.74 (0.60–0.83)
Physical symptoms (3)	0.60	0.80	0.80 (0.69–0.87)
Psychological function PSD(11)	0.83	0.91	0.91 (0.86–0.95)
Cognition (2)	0.53	0.83	0.82 (0.72–0.89)
Anxiety (3)	0.69	0.83	0.83 (0.74–0.89)
Depression (3)	0.79	0.90	0.90 (0.83–0.94)
Self-consciousness (3)	0.70	0.94	0.94 (0.90–0.96)
Social function SOD(11)	0.62	0.77	0.77 (0.65–0.86)
Social support/security (6)	0.73	0.84	0.84 (0.75–0.90)
Social Effects (4)	0.63	0.74	0.73 (0.59–0.83)
Sexual Function (1)	–	0.84	0.84 (0.74–0.90)
Specific domain SPD (14)	0.73	0.80	0.80 (0.69–0.87)
Upper abdomen pain(4)	0.44	0.72	0.72 (0.57–0.82)
Acid regurgitation/salivation(2)	0.35	0.86	0.86 (0.77–0.91)
Hiccup (1)	–	0.84	0.84 (0.75–0.90)
Flatulent (1)	–	0.83	0.83 (0.73–0.89)
Changing in stool habit (1)	–	0.77	0.77 0.64–0.85
Effects of mental and life(5)	0.63	0.73	0.72 (0.58–0.82)
Total TOT(44)	–	0.89	0.89 (0.82–0.93)

– not acceptable/suitable, ICC: intra-class correlation

In the second evaluation (two-day follow-up), data from 63 patients were used for test–retest reliability analysis. The test–retest correlation coefficients for the 4 domains and the overall were larger than 0.80 except for SOD (0.77), while they were ranging between 0.72–0.94 at facets level. The ICC result calculated according to the definition of absolute consistency is very similar to the Pearson correlation coefficient.

### Results from generalizability theory

G-Studies were performed to estimate the variance components for four domains of the QLICD-PU (see Table 4), in which 153 patients filled out this QOL instrument with 44 items.

**Table 4:** The estimated variance components and percentage of variance accounted for by effects (percent) for p × i design in G-study for four domains of quality of life instrument QLICD-PU (${\text{n}}_{{\text{p}}}^{{\prime }} = 153$)

Domain	*p*(person)		*i*(item)		p* i(person*item)	
	Variance component	Percent (%)	Variance component	Percent (%)	Variance component	Percent (%)
PHD (${\text{n}}_{{\text{i}}}^{{\prime }} = 8$)	0.517	27.80	0.224	12.04	1.119	60.16
PSD (${\text{n}}_{{\text{i}}}^{{\prime }} = 11$)	0.547	35.92	0.128	8.40	0.848	55.68
SOD (${\text{n}}_{{\text{i}}}^{{\prime }} = 11$)	0.190	12.20	0.092	5.91	1.275	81.89
SPD (${\text{n}}_{{\text{i}}}^{{\prime }} = 14$)	0.143	8.99	0.257	16.15	1.191	74.86

PHD: physical domain, PSD: psychological domain, SOD: social domain, SPD: specific domain,

p: person effect, i: item effect, p × i: person-by-item interaction effect

As can be seen from Table 4, for the four domains of physical, psychological, social and the specific, the largest source of variation were due to person-by-item interactions ranging from 55.68% to 81.89%, while variances accounted for by person were the second for three domains of physical, psychological and social ranging from 12.20% to 35.92%.

The D-Studies were performed to estimate the Generalizability coefficient (G-coefficient) and index of dependability (Ф coefficient) for four domains of the QLICD-PU for the p × i current design (physical domain includes 8 items, psychological domain includes 11 items**,** social domain includes 11 items and the specific domain includes 14 items), as well as the alternative designs with varied numbers of items (see Table 5).

**Table 5:** G-coefficients and Ф-coefficients for different numbers of items for p × I design in D-study for four domains of quality of life instrument QLICD-PU

Domain	Number of items	$\sigma^{2} ({\text{P}})$	$\sigma^{2} ({\text{I}})$	$\sigma^{2} ({\text{PI}})$	$\sigma^{2} (\delta )$	$\sigma^{2} (\Delta )$	$\sigma^{2} ({\text{X}}_{{{\text{PI}}}} )$	${\text{E}}\rho^{2}$	$\Phi$
Physical domain	6	0.517	0.037	0.186	0.186	0.224	0.042	0.735	0.698
	**8**	**0.517**	**0.028**	**0.140**	**0.140**	**0.168**	**0.032**	**0.787**	**0.755**
	9	0.517	0.025	0.124	0.124	0.149	0.029	0.806	0.776
	11	0.517	0.020	0.102	0.102	0.122	0.024	0.836	0.809
Psychological domain	9	0.338	0.010	0.095	0.095	0.105	0.013	0.780	0.763
	**11**	**0.338**	**0.007**	**0.069**	**0.069**	**0.076**	**0.010**	**0.830**	**0.815**
	13	0.338	0.006	0.059	0.059	0.065	0.009	0.852	0.839
	15	0.338	0.005	0.051	0.051	0.056	0.008	0.869	0.858
Social domain	9	0.190	0.010	0.142	0.142	0.152	0.012	0.573	0.556
	**11**	**0.190**	**0.008**	**0.116**	**0.116**	**0.124**	**0.010**	**0.622**	**0.605**
	13	0.190	0.007	0.098	0.098	0.105	0.009	0.660	0.644
	16	0.190	0.006	0.080	0.080	0.085	0.008	0.705	0.690
	17	0.190	0.005	0.075	0.075	0.080	0.007	0.717	0.703
	27	0.190	0.003	0.047	0.047	0.051	0.005	0.801	0.790
	29	0.190	0.003	0.044	0.044	0.047	0.005	0.812	0.801
Specific domain	11	0.143	0.023	0.108	0.108	0.132	0.025	0.568	0.520
	**14**	**0.143**	**0.018**	**0.085**	**0.085**	**0.103**	**0.020**	**0.626**	**0.580**
	17	0.143	0.015	0.070	0.070	0.085	0.016	0.671	0.626
	20	0.143	0.013	0.060	0.060	0.072	0.014	0.705	0.663
	24	0.143	0.011	0.050	0.050	0.060	0.012	0.742	0.703
	34	0.143	0.008	0.035	0.035	0.043	0.009	0.803	0.770
	41	0.143	0.006	0.029	0.029	0.035	0.007	0.831	0.802

Item number for present scale is shown in bold

$\sigma^{2} (\delta )$ is the variance components of relative error, $\sigma^{2} (\Delta )$ is the variance components of absolute error, $\sigma^{2} ({\text{X}}_{{{\text{PI}}}} )$, is the variance components of error when estimating the universe score by using sample mean, ${\text{E}}\rho^{2}$ is the Generalizability coefficient, $\Phi$ is the index of dependability

### Responsiveness

The data from 135 patients who completed the questionnaire after treatments were used to assess responsiveness. The paired t-test and the response index SRM were used to check the average score change of each domain/facet of QLICD-PU before and after treatments. The results are shown in Table 6. It can be seen that except for social impact and sexual function, all domains/facets and overall scale have undergone major changes (*P* < 0.01), with SRM ranging from 0.04 to 1.03 and domain-level SRM from 0.34 to 1.03.

**Table 6:** Responsiveness of the quality of life instrument QLICD-PU (n = 135)

Domains/facets (number of items)	Before treatment Mean SD		After treatment Mean SD		Differences mean SD		*t*	*p*	SRM
	Mean	SD	Mean	SD	Mean	SD			
Physical Function (8)	58.70	19.53	71.57	13.06	− 12.87	17.37	− 8.61	< 0.001	0.74
Independence (3)	71.23	27.41	82.35	16.48	− 11.11	23.39	− 5.52	< 0.001	0.47
Appetite and Sleep (2)	43.24	25.59	58.33	21.87	− 15.09	24.39	− 7.19	< 0.001	0.62
Physical Symptoms (3)	56.48	22.11	69.63	15.75	− 13.15	21.07	− 7.25	< 0.001	0.62
Psychological function (11)	75.35	16.15	83.84	13.85	− 8.48	11.78	− 8.37	< 0.001	0.72
Cognition (2)	68.61	22.85	81.11	17.80	− 12.50	23.76	− 6.11	< 0.001	0.53
Anxiety (3)	69.57	22.24	84.81	17.57	− 15.25	20.09	− 8.82	< 0.001	0.76
Depression (3)	82.47	20.70	87.35	14.71	− 4.88	14.27	− 3.97	< 0.001	0.34
Self-Consciousness (3)	78.52	19.09	81.17	16.51	− 2.65	10.95	− 2.82	0.006	0.24
Social function (11)	67.41	14.21	71.16	12.92	− 3.75	10.99	− 3.97	< 0.001	0.34
Social Support/Security (6)	69.69	19.73	76.60	15.04	− 6.91	13.64	− 5.89	< 0.001	0.51
Social Effects (4)	64.44	22.08	63.75	21.27	0.69	18.00	0.45	0.655	0.04
Sexual Function (1)	65.56	30.67	68.15	27.90	− 2.59	21.22	− 1.42	0.158	0.12
Sub-total (QLICD-GM) (30)	68.00	12.56	75.92	10.90	− 7.92	10.02	− 9.19	< 0.001	0.79
Specific domain (14)	62.72	11.86	74.44	12.14	− 1.72	11.43	− 11.91	< 0.001	1.03
Upper abdomen pain(4)	53.47	17.90	68.43	11.66	− 14.95	19.60	− 8.86	< 0.001	0.76
Acid regurgitation/salivation(2)	75.83	21.28	86.94	18.19	− 11.11	19.77	− 6.53	< 0.001	0.56
Hiccup (1)	70.00	28.78	85.56	19.19	− 15.56	26.43	− 6.84	< 0.001	0.59
Flatulent (1)	60.19	29.97	80.00	23.22	− 19.81	27.67	− 8.32	< 0.001	0.72
Changing in stool habit (1)	48.89	28.95	54.63	24.66	− 5.74	27.14	− 2.46	0.014	0.21
Effects of mental and life(5)	66.70	18.66	74.89	19.24	− 8.19	13.90	− 6.84	< 0.001	0.59
Total (44)	66.32	10.73	75.45	10.36	− 9.13	9.38	− 11.31	< 0.001	0.97

SRM: Standardized response mean

## Discussions

The focus of this study was to develop and validate a special QOL instrument QLICD-PU for peptic ulcer disease. We used a modular method that combines a general module with a specific module for a specific disease to capture common features within the disease category and the differences between the specific diseases [18–20]. In fact, we have developed a new instrument system for chronic diseases (QLICD) systematically and effectively by adopting this modular approach, in which the general module QLICD-GM is used for various chronic diseases, and QLCID-PU is only for a specific scale of PUD. This method uses the same general module and similar structure to unify all QLICD specific disease tools.

Compared with existing instruments, QLICD-PU has several advantages [20–23]. First, it can compare the QOL of various diseases through a general module, and can also capture symptoms and side effects through a specific module. For example, we can use QLICD-GM to capture general QOL in patients with different diseases, while we can also employ QLICD-PU and QLICD-CG to capture differences in QOL in PUD and chronic gastritis patients further. Secondly, the different mean scores can be calculated to detect detailed changes, not only at the domain level (4 domains) but also at the facet level (16 facets), because the QLICD-PU is consisted of a moderate number of items with a clear hierarchy (item → facet → domain → the overall). Users can choose one or two levels for research at their convenience. Third, the most important value of QLICD-PU is the profound Chinese cultural background behind it. For example, Chinese culture focuses on family relations and pedigree, diet, temperament, and noble spirit, all of which are reflected in QLICD-PU through items that focus on appetite, sleep, energy, and family support. The English language version of the QLICD-PU was also as a supplementary file in order to more researchers can learn this instrument.

Generally speaking, practical QOL instruments require excellent psychometric characteristics, including validity, reliability and responsiveness. Validity is the degree to which the tool can capture what it claims to measure. Follow WHO's definition of quality of life [34] and systematic development procedure, we developed QLICD-PU for PUD patients through focus group discussions, in-depth interviews, and pre-tests to effectively reduce the number of items. It has been reduced effectively the number of items to 30 from an initial 73 item bank for the final version of the general module [20], and reduced to 14 items of the specific module from the first 29 items. This process helps us achieve good content validity and conceptual structure of this instrument. Correlation and factor analysis were used to confirm the construct validity. Correlation analysis showed that the relationship between items and their domains/facets is strong, but the relationship between items and other domains/facets is weak. Factor analysis showed that the components extracted from the data are consistent with the theoretical structural framework of the instrument. These results confirmed evidence supporting the good construct validity. The correlation coefficients between the QLICD-PU and SF-36 domain scores demonstrated that the criterion-related validity and construct validity (the convergent and divergent validity) are both high.

Reliability refers to the repeatability or consistency of item ratings in different assessments. In this study, internal consistency reliability (Cronbach's α), test–retest reliability (Pearson r) and ICC were applied. Our results showed that the internal consistency coefficients for the QLICD-PU domains and the overall are both greater than 0.70 except for the social function domain (0.62). The test–retest reliability coefficient of the overall score is 0.89, while the test–retest reliability coefficients of domains are greater than 0.80 (except for social function domain) (0.77). Taking into account that the internal consistency coefficient should be greater than 0.70 and the test–retest reliability coefficient should be greater than 0.80, which are considered satisfactory, these results indicated that the instrument has good reliability overall.

Responsiveness (sensitivity to detect changes) is the most important desirable characteristic of the QOL scale in clinical applications. There are two types of assessment methods: internal and external [28, 29]. In this study, we used a paired t-test to focus on internal responses to compare the average response before and after treatments. We used SRM as a responsiveness indicator, with 0.20, 0.50, and 0.80 representing small, moderate, and large responsiveness [28, 29]. The QOL scores had significant changes after treatments for all domains and the overall score (*P* < 0.001) with SRM being greater 0.70 exception of the social function domain (0.34), suggesting QLICD-PU has good responsiveness.

In addition to classical test theory analysis, this study also applied generalization theory. This research presented both G-coefficients and *Ф*, and also their changes when items assumed to be changing. For the physical and psychological domains, we estimated a G-coefficients of 0.787, 0.830 and index of dependability of 0.755, 0.815 respectively for the current design. It can be considered that it meets the 0.70 standards. For the social domain, the current design G-coefficient is estimated to be 0.622, and the index of dependability is 0.605, which is lower than the acceptable 0.70. Therefore, the items of this domain need to be improved. For an alternative design with 17 items, the G coefficient is estimated to be 0.717 and the index of dependability is 0.703, which will satisfy acceptable reliability. For the specific domain, the G-coefficient of the current design is estimated to be 0.626, and the index of dependability is 0.580, which is also lower than the acceptable 0.70. Similarly, the G-coefficient estimated to be 0.742 and the index of dependability 0.703 when an alternative design with 24 items. Therefore, these analyses suggested that the number of items of the social domain needs to be increased from 11 to 17, and the specific domain from 11 to 24 to reach acceptable reliability. However, it may not be practical to increase the length of the test in practice, as reliability is reduced if the subject is required to complete too many items at the same time. Researchers or instrument users can decide to add items or tolerate reasonable low reliability.

## Conclusions

In conclusion, the QLICD-PU can be used as a useful tool for assessing the quality of life of patients with PUD, with good psychometric characteristics and many advantages. The analysis from Generalizability theory not only confirmed the reliability of the scale as a whole, but it also provided more information than CTT. However, the number of items for social and the specific domains should be increased to increase reliability. Besides, the quality of items in these 2 domains should also be addressed.

## Supplementary information

**Additional file 1.** The questionnaire was developed for this study, the English language version was as asupplementary file.

## Data Availability

The data (two formats: SPSS and Excel) can be available by request from Prof. Chonghua Wan (Email: wanchh.hotmail.com).

## Abbreviations

CTT: Classical test theory
D-study: Decision study
FDDQL: Functional digestive disorder quality of life questionnaire
G-study: Generalizability study
GSRS: Gastrointestinal Symptom Rating Scale
GT: Generalizability theory
HRQOL: Health-related quality of life
ICC: Intra-class correlation
PGWB: Psychological general well-being
PUD: Peptic ulcer disease
QLDUP: Quality of life in duodenal ulcer patients
QLICD: Quality of life instruments for chronic diseases
QOL: Quality of life
QOLRAD: Quality of life in reflux and dyspepsia;
QPD: Quality of life in peptic disease;
SRM: Standardized response mean
