# Supplementary material for: Development and validation of the peptic ulcer scale under the system of quality of life instruments for chronic diseases based on classical test theory and generalizability theory
Source: BMC Gastroenterol. 2020 Dec 14;20:422. doi: 10.1186/s12876-020-01562-y (PMC7734778; doi:10.1186/s12876-020-01562-y)
Supplement: Supplementary file 1 — Additional file 1. The questionnaire was developed for this study, the English language version was as asupplementary file. [file 12876_2020_1562_MOESM1_ESM.doc]

**Quality of Life Instruments for Chronic Diseases – Peptic Ulcer Scale**

**QLICD-PU (V1.0)**

***INSTRUCTION:*** This questionnaire helps doctor to know your feeling about your health situation in last week. Your answers will help the doctor choosing treatment and rehabilitation appropriately. There is no right or wrong in your answers. Please read the following questions carefully, and mark the number with circle which most fitting your situation according to your own standard or feeling. You can choose the answer that is closest to your true feeling in the case that you are not sure how to answer some question. The information that you provide will remain strictly confidential.

**Physical Function**

|  |  | Not at little | A little | Moderately | Very much | Extremely |
| --- | --- | --- | --- | --- | --- | --- |
| PH1 | Could you take care of your daily life? (e.g., eating, dressing, washing, using toilet）? | 1 | 2 | 3 | 4 | 5 |
| PH2 | Have you felt fatigue easily? | 1 | 2 | 3 | 4 | 5 |
| PH3 | Do you have any trouble walking 800m or more？ | 1 | 2 | 3 | 4 | 5 |
| PH4 | Do you have any trouble going up and down stairs？ | 1 | 2 | 3 | 4 | 5 |
| PH5 | Have you need to take medication to maintain daily activities？ | 1 | 2 | 3 | 4 | 5 |
| PH6 | Have you had a good appetite? | 1 | 2 | 3 | 4 | 5 |
| PH7 | Were you satisfied with your sleep? | 1 | 2 | 3 | 4 | 5 |
| PH8 | Have you felt pain or uncomfortable? | 1 | 2 | 3 | 4 | 5 |

**Psychological Function**

|  |  | Not at little | A little | Moderately | Very much | Extremely |
| --- | --- | --- | --- | --- | --- | --- |
| PS1 | Have your memory and concentration been affected by the disease？ | 1 | 2 | 3 | 4 | 5 |
| PS2 | Have you felt mentally miserable because of the disease? | 1 | 2 | 3 | 4 | 5 |
| PS3 | Have you felt lonely and helpless? | 1 | 2 | 3 | 4 | 5 |
| PS4 | Have you felt pessimism and despair？ | 1 | 2 | 3 | 4 | 5 |
| PS5 | Have you been worried about your disease？ | 1 | 2 | 3 | 4 | 5 |
| PS6 | Have you felt fretful or irritable？ | 1 | 2 | 3 | 4 | 5 |
| PS7 | Have you felt nervous and anxious? | 1 | 2 | 3 | 4 | 5 |
| PS8 | Is there any possibility for you to terminate taking drug because of its side effects? | 1 | 2 | 3 | 4 | 5 |
| PS9 | Have you thought yourself as the burden of the family? | 1 | 2 | 3 | 4 | 5 |
| PS10 | Have you felt self-abasement because of your disease? | 1 | 2 | 3 | 4 | 5 |
| PS11 | Have you covered the emotions, but could not forget? | 1 | 2 | 3 | 4 | 5 |

**Social Function**

|  |  | Not at little | A little | Moderately | Very much | Extremely |
| --- | --- | --- | --- | --- | --- | --- |
| SO1 | Has the disease or treatments interfered with your work or housework? | 1 | 2 | 3 | 4 | 5 |
| SO2 | Could you undertake appropriate family roles (such as parents, husband or wife)? | 1 | 2 | 3 | 4 | 5 |
| SO3 | Have you decreased your caring and attention to the families because of the disease? | 1 | 2 | 3 | 4 | 5 |
| SO4 | Have you had good relations with your families? | 1 | 2 | 3 | 4 | 5 |
| SO5 | Could you acquire material and emotional help and support from your family when you need? | 1 | 2 | 3 | 4 | 5 |
| SO6 | Has the disease affected you participating leisure activities which you like? | 1 | 2 | 3 | 4 | 5 |
| SO7 | Could you treat the illness positively and optimistically? | 1 | 2 | 3 | 4 | 5 |
| SO8 | Have you thought that the treatments you received was good for curing the disease？ | 1 | 2 | 3 | 4 | 5 |
| SO9 | Has the economic problems caused by illness or treatment affected your life？ | 1 | 2 | 3 | 4 | 5 |
| SO10 | Could you get the care and support from your friends and relatives? | 1 | 2 | 3 | 4 | 5 |
| SO11 | Has the disease or treatment affected your sexual activities？ | 1 | 2 | 3 | 4 | 5 |

**Specific Module**

|  |  | Not at little | A little | Moderately | Very much | Extremely |
| --- | --- | --- | --- | --- | --- | --- |
| PU1 | Did you have pain (sore or blunt painful, burning pain, swelling) in epigastria? | 1 | 2 | 3 | 4 | 5 |
| PU2 | Did you have heartburn（noisy） feeling in epigastria? | 1 | 2 | 3 | 4 | 5 |
| PU3 | Did you have pain or discomfort in abdomen at night or at hungry? | 1 | 2 | 3 | 4 | 5 |
| PU4 | Would your upper abdomen pain/ uncomfortable feelings be relieved after dinner? | 1 | 2 | 3 | 4 | 5 |
| PU5 | Did you have acid regurgitation? | 1 | 2 | 3 | 4 | 5 |
| PU6 | Did you have any belch (burps)? | 1 | 2 | 3 | 4 | 5 |
| PU7 | Did you feel abdominal distension？ | 1 | 2 | 3 | 4 | 5 |
| PU8 | Did you salivate (flow saliva)? | 1 | 2 | 3 | 4 | 5 |
| PU9 | Did your move bowels condition normal? | 1 | 2 | 3 | 4 | 5 |
| PU10 | Did you feel upset or distress because of need making gastroscopy inspection? | 1 | 2 | 3 | 4 | 5 |
| PU11 | Did you feel vexed because can’t eat certain favorite food/drink (such as acrimony, acidic, sweet, strong tea, liquor, etc.) for gastropathy? | 1 | 2 | 3 | 4 | 5 |
| PU12 | Were your life made in troubled/limit by dine at fix time? | 1 | 2 | 3 | 4 | 5 |
| PU13 | Were you worried about that the sores may cause more severe disease (obstruction, punch or cancerous)? | 1 | 2 | 3 | 4 | 5 |
| PU14 | Did you feel vexed because of often taking stomach medications to prevent ulcer recurrence? | 1 | 2 | 3 | 4 | 5 |
